# HLA-B*5701 testing to predict abacavir hypersensitivity

**DOI:** 10.1371/currents.RRN1203

**Published:** 2010-12-07

**Authors:** Joseph D. Ma, Kelly C. Lee, Grace M. Kuo

**Affiliations:** ^*^University of California, San Diego, Skaggs School of Pharmacy & Pharmaceutical Sciences and ^†^University of California, San Diego, Skaggs School of Pharmacy and Pharmaceutical Sciences

## Abstract

Abacavir is a nucleoside reverse transcriptase inhibitor used for combination antiretroviral therapy for treating human immunodeficiency virus (HIV) infection. An adverse effect from abacavir is a treatment-limiting hypersensitivity reaction, which can be severe and potentially life-threatening. Abacavir-induced hypersensitivity reaction has been associated with the presence of the major histocompatibility complex class I allele HLA-B*5701. A screening test for the HLA-B*5701 allele can assist clinicians to identify patients who are at risk of developing a hypersensitivity reaction to abacavir.

## **Clinical Scenario**

Abacavir hypersensitivity reaction affects 5 to 8% of patients and can be observed during the first 6 weeks of antiretroviral therapy [1] [2].  Symptoms of an abacavir hypersensitivity reaction include skin rash, fever, malaise, gastrointestinal symptoms, and respiratory symptoms.  Severe forms of the skin rash may result in Stevens-Johnson Syndrome, toxic epidermal necrolysis, or systemic lupus erythematosus [3].  If a patient experiences a hypersensitivity reaction, abacavir is discontinued and symptoms generally resolve within 72 hours [4]. Restarting abacavir is contraindicated as it can result in a potentially life-threatening reaction and even death [5] [6] [7] [8].  The HLA-B*5701 screening test minimizes potential toxicities to abacavir by identifying patients who may be at risk of developing a hypersensitivity reaction.   

##  **Test Description**

Sequence-based genotyping and polymerase chain reaction (PCR) sequencing of specific oligonucleotide probes are the most widely used techniques.  To test for the HLA-B*5701 allele, a blood or saliva specimen is collected.  The genetic sequences coding for the *HLA* -B*5701 are probed and reported as positive if the allele is present, or negative if the allele is absent.  

## **Public Health Importance**

There are approximately 33 million people worldwide who are living with HIV/AIDS [9]; among them are approximately 1.2 million Americans, with an estimated 56,300 newly diagnosed infections each year.  The Centers for Disease Control and Prevention (CDC) estimates that 21% of HIV-positive people are unaware that they are infected [10].  Combination antiretroviral therapy is the most effective pharmacotherapy for HIV treatment [10].  Minimizing adverse effects of antiretroviral therapy is critical to controlling the infection and maintaining treatment adherence.

## **Published Reviews, Recommendations and Guidelines** 

**Systematic evidence reviews**

A technology report based on research conducted by the Tufts Medical Center Evidence-based Practice Center under contract to the Agency for Healthcare Research and Quality (AHRQ) has been published (Project ID: GEND0508; March 2010).  Genetic tests for non-cancer diseases/conditions, including abacavir were detailed [11]. 

**Recommendations by independent group**

Screening for HLA-B*5701 prior to initiation of abacavir is recommended by the U.S. Department of Health and Human Services  (DHHS) Panel on Antiretroviral Guidelines for Adults and Adolescents (A Working Group of the Office of AIDS Research Advisory Council) [12] and the Panel on Antiretroviral Therapy and Medical Management of HIV-infected children [13].

Pediatric, adolescent, and adult patients testing positive for the HLA-B*5701 allele should not be prescribed abacavir [12] [13].

**Guidelines by professional groups**

The Infectious Diseases Society of America (IDSA) has issued clinical guidelines which state “HLA-B*5701 testing should be performed prior to initiating abacavir therapy to reduce the risk of a hypersensitivity reaction.  Patients who are positive for the HLA-B*5701 haplotype should not be treated with abacavir” [14].

**Other groups**

In July 2008, the U.S. Food and Drug Administration (FDA) issued a post-marketing communication [15] to update the prescribing information for abacavir.  The updated black box warning stated “Prior to initiating therapy with abacavir, screening for the HLA-B*5701 allele is recommended; this approach has been found to decrease the risk of hypersensitivity reaction” [16]. 

## **Evidence Overview** 

***Analytic Validity*:**Test accuracy and reliability in measuringthe HLA-B*5701 allele (analytic sensitivity and specificity).

Among 4 international laboratories, analytic specificity of detecting the HLA-B*5701 allele via PCR sequencing was 100% [17].  Analytic sensitivity ranged from 99.4% - 100%, with 1 laboratory reporting a single false-negative [17].  Given these data, there appears to be very little variability for the analytic specificity and sensitivity.

***Clinical Validity*:**Test accuracy and reliability in predicting abacavir hypersensitivity (predictive value). 

The prevalence of the HLA-B*5701 allele is highest in Caucasian populations (5-8%) [3] [18] [19] [20].  In African-American, Asian, and Hispanic populations, the prevalence is 0.26-3.6% [19] [20] [21] [22].  In a review of the adult and adolescent antiretroviral guidelines and the abacavir prescribing information [12] [16], the prevalence of the HLA-B*5701 allele between ethnic populations has no impact on clinical recommendations. 

In studies conducted in North America, Europe, and Australia where patients were diagnosed with an abacavir hypersensitivity reaction based on symptom presentation, HLA-B*5701 test sensitivity was 46-78% [22] [23] [24].  In contrast, HLA-B*5701 test sensitivity was 94-100% in patients with an immunologically confirmed (via skin patch testing) abacavir hypersensitivity reaction [25] [26] [27].  There is suggestion that the discrepancy of lower estimates of test sensitivity was the inclusion of non-abacavir related hypersensitivity reactions [28].  

HLA-B*5701 test specificity, regardless of whether the abacavir hypersensitivity reaction is based on symptom presentation or immunologic confirmation, is 90-100% [22] [23] [24] [25] [26] [27]. 

Pooled data from 3 study populations reported a positive predictive value and negative predictive value of 82% (95% Confidence Interval [CI] 71-90%) and 85% (95% CI 81-88%), respectively [22] [23] [24].   

A report by Hughes et al. suggested a “high genetic penetrance of HLA-B*5701 in predisposing [patients] to abacavir hypersensitivity" [24].

***Clinical Utility*: **Net benefit of test in improving health outcomes

The PREDICT-1 study was a double-blind, prospective, randomized study of 1,956 patients from 19 countries. The incidence of confirmed abacavir hypersensitivity was 2.7% in the control group versus 0% in the HLA-B*5701 screened group (*p*<0.001) [25].  In another prospective study of 137 patients of an ethnically mixed French HIV population, the incidence of an abacavir hypersensitivity in the HLA-B*5701 screened group was 0% [29].

The ARIES study was an open-label, multicenter, North American study of 725 patients.  Patients who were HLA-B*5701 negative had less than 1% clinically suspected abacavir hypersensitivity, and none had positive skin patch tests at 30 weeks [30].

HLA-B*5701 testing to prevent abacavir hypersensitivity has been reported to be cost-effective [24] [31].  In one study, HLA-B*5701 testing resulted in a cost-effectiveness ratio of $36,000 per quality-adjusted life expectancy compared to no testing [31].

Educating/training of hospital staff, monitoring, and implementing facility services for HLA-B*5701 testing have been reported by several institutions [21] [32] [33]. 

##  **Links**

- AIDS.gov
- AIDSinfo
- Pharmacogenomics Knowledge Base (PharmGKB)
- U.S. Food and Drug Administration. Table of Valid Genomic Biomarkers in the Context of Approved Drug Labels

## **Acknowledgments**

We acknowledge Sara Bedrosian, BA, BFA and William D. Dotson, PhD from the CDC for reviewing this document and providing comments.

## **Funding information**

This project was funded in part by the CDC Cooperative Agreement #1U38GD000070, Pharmacogenomics Education Program (PharmGenEd™): Bridging the Gap between Science and Practice.  The contents of this manuscript are solely the responsibility of the authors and do not necessarily represent the official views of CDC.

## **Competing interests**

The authors have declared that no competing interests exist.
